# Role of Alkylamines in Tuning the Morphology and Optical Properties of SnS_2_ Nanoparticles Synthesized by via Facile Thermal Decomposition Approach

**DOI:** 10.3390/nano12223950

**Published:** 2022-11-09

**Authors:** Rama Gaur, Syed Shahabuddin, Irfan Ahmad, Nanthini Sridewi

**Affiliations:** 1 Department of Chemistry, School of Technology, Pandit Deendayal Energy University, Knowledge Corridor, Raysan, Gandhinagar 382426, Gujarat, India; 2 Department of Clinical Laboratory Sciences, College of Applied Medical Sciences, King Khalid University, Abha 61421, Saudi Arabia; 3 Department of Maritime Science and Technology, Faculty of Defence Science and Technology, National Defence University of Malaysia, Kuala Lumpur 57000, Malaysia

**Keywords:** SnS_2_ nanoparticles, thermal decomposition, nanoflakes, nanoflowers, capping agent, alkylamines

## Abstract

The present study reported the synthesis of SnS_2_ nanoparticles by using a thermal decomposition approach using tin chloride and thioacetamide in diphenyl ether at 200 °C over 60 min. SnS_2_ nanoparticles with novel morphologies were prepared by the use of different alkylamines (namely, octylamine (OCA), dodecylamine (DDA), and oleylamine (OLA)), and their role during the synthesis was explored in detail. The synthesized SnS_2_ nanostructures were characterized using an array of analytical techniques. The XRD results confirmed the formation of hexagonal SnS_2_, and the crystallite size varied from 6.1 nm to 19.0 nm and from 2.5 to 8.8 nm for (100) and (011) reflections, respectively. The functional group and thermal analysis confirmed the presence of organics on the surface of nanoparticles. The FE-SEM results revealed nanoparticles, nanoplates, and flakes assembled into flower-like morphologies when dodecylamine, octylamine, and oleylamine were used as capping agents, respectively. The analysis of optical properties showed the variation in the bandgap and the concentration of surface defects on the SnS_2_ nanoparticles. The role of alkylamine as a capping agent was explored and discussed in detail in this paper and the mechanism for the evolution of different morphologies of SnS_2_ nanoparticles was also proposed.

## 1. Introduction

The growing population around the world arouses energy concerns in all kinds of fields. It took us a long time to realize that “the ultimate source of energy sun is the ultimate source”. The latest estimates by scientists prove that the amount of solar energy the sun gives to the earth in a single day is sufficient to meet the total energy needs of the world for 27 years at the current rate of consumption [1]. We only have to harness a very little fraction of it to meet all our energy needs for all time to come. Extensive research has been made over the years on solar cells to improve their efficiency and augment their commercialization. Hence, a continuous effort has been made in this area and is still being made to achieve maximum efficiency by modulating the materials used as the photoanode. Reports are available on the use of metal selenides as sensitizers and electron acceptors in dye-sensitized solar cells with an enhanced power conversion efficiency of up to 9.49% [2,3]. Owing to the negative traits such as toxicity and instability of selenides, we need to look for alternative inorganic materials as electron acceptors for future solar cells. The use of layered metal sulfides such as MoS_2_, SnS_2_, and WS_2_ is considered a potential candidate for this purpose [4]. SnS_2_ is a moderate band gap semiconductor (2–2.5 eV) with a layered structure. It has been reported to exhibit a CdI_2_-type structure, where hexagonally ordered planes of Sn atoms are held between two hexagonally ordered planes of S atoms, and with adjacent sulfur layers [5,6]. It possesses excellent optical and electrical properties and is an important material for optoelectronic devices. Hence, SnS_2_ is considered a potential candidate for various applications such as catalysis, solar cells, sensors, photodetectors, lithium and sodium ion batteries, light-emitting diodes, etc. [4,5,6,7,8].

Looking at the synthetic aspects and environmental concerns, SnS_2_ is comparatively easy to synthesize and has no toxic effects on the environment. Various methods have been reported for the synthesis of SnS_2_ nanoparticles, such as hydrothermal, sol–gel, laser ablation, chemical vapor deposition, etc. [4,9,10,11]. The reported synthetic approaches involve harsh conditions, high temperatures, long reaction times, and a lack of control over the shape and size of the materials. Alternate approaches such as solvent-assisted thermal decomposition allow the easy and facile synthesis of shape- and size-controlled nanomaterials.

Morphology-dependent studies on nanomaterials have revealed that the same material with different morphologies exhibits significantly different properties [12,13]. Research is being conducted in this direction to tune the morphology and surface characteristics by chemical methods. Researchers have reported different morphologies of SnS_2_ such as flakes, nanosheets, worm-like shapes, nanorods, flower-like shapes, nanobelts, etc. [14,15,16,17,18,19].

Alkylamines have been reported to be an important class of stabilizers during the synthesis of colloidal semiconductor nanocrystals. A series of alkylamines have been reported as stabilizer/capping agents for the preparation of group II–VI and IV–VI nanomaterials [20]. The addition of these alkylamines during the synthesis of semiconductor nanocrystals aids nucleation and affects crystal growth [21]. The role of alkylamines in controlling morphological characteristics has always been debatable. A few reports suggest that the addition of alkylamine retards the kinetics by passivating the crystal surface, hence retarding crystal growth [22,23]. On the other hand, a few reports suggest their role as promoters by enhancing the nucleation kinetics and influencing crystal growth [24,25,26]. The above reports point towards the chemical interaction of alkylamines with precursor molecules, resulting in a pre-conditioned molecular precursor. Alkylamine-substituted precursors are decomposed to form nanocrystals with defined morphologies and unique optical and structural properties. The preferential adsorption of alkylamines on the surface of nanocrystals results in the accelerated growth of the other planes. The mechanistic insights into the role of alkylamines have been discussed by García-Rodríguez et al. (2014) in their reports [20]. The interaction of alkylamines with the growing crystal is a temperature-dependent phenomenon. The amine adsorption is minimum at low temperatures and is increased with an increase in the reaction temperature. Li et al. (2004) and Pradhan et al. (2007) suggested that alkylamines activate the precursors of ZnSe, ZnS, and CdSe nanocrystals at various reaction temperatures [25,27]. Similarly, Sun et al. reported that dodecylamine increases the rate of consumption of phosphine selenide precursor as well as the rate of CdSe nanocrystal growth [26]. In contrast, Guo et al. suggested that alkylamines decrease their reactivity instead [28]. Mourdikoudis et al. have reviewed oleyamine as a solvent, surfactant, and reducing agent, for the controlled preparation of a wide range of nanomaterials including metal oxides, metal sulfides, noble metals, and alloy nanocrystals [29]. These unique effects of alkylamines significantly improve the aspect of controlled morphology with desired properties.

From the above discussion, it is very clear that alkylamines play an important role during the synthesis by controlling crystal growth. The present paper aimed at exploring the role of alkylamines as a capping agent and their effect on the optical properties and the morphology of SnS_2_ nanoparticles. In a continuation of previous work, the authors attempted the synthesis in the presence of different alkylamines (oleylamine, octylamine, and dodecylamine).

## 2. Experimental Section

### 2.1. Reagents

Tin(IV) chloride pentahydrate, thioacetamide (Sigma Aldrich^®^ 99%, Ahemdabad, Gujarat, India), diphenyl ether (Sigma Aldrich^®^ 99%, Ahemdabad, Gujarat, India), octylamine (Sigma Aldrich^®^ 99%, Ahemdabad, Gujarat, India), oleylamine (Sigma Aldrich^®^ 99%, Ahemdabad, Gujarat, India), dodecylamine (Sigma Aldrich^®^ 99%, Ahemdabad, Gujarat, India), and Millipore^®^ water were acquired. All the chemicals were used as received. Methanol used during the reaction was distilled before use.

### 2.2. Synthesis of SnS_2_ Nanoparticles

The SnS_2_ nanoparticles were synthesized using a simple thermal decomposition approach. In a typical synthesis, 1 mmol of SnCl_4_·5H_2_O and 1 mmol of CH_3_CSNH_2_ were added to 10 mL of diphenyl ether in a 50 mL round-bottom flask and were refluxed at 200 °C in the air for 1 h. After the completion of the reaction, a slurry was obtained and cooled to room temperature. A total of 30 mL of methanol was added to the slurry and the precipitate obtained was washed using an excess of methanol. The precipitate was dried overnight at 65 °C under a vacuum. For the preparation of nanoparticles in the presence of capping agents (1 mmol), oleylamine, octylamine, and dodecylamine were added to different reaction setups along with the Sn and S precursors to diphenyl ether in the initial step. The synthetic details and nomenclature of the SnS_2_ samples, prepared in the present study, are given in Table 1.

### 2.3. Characterization

The as-prepared SnS_2_ samples were characterized using an array of sophisticated characterization techniques. The SnS_2_ nanostructure was analyzed for structural, compositional, and morphological characterization. The phase analysis was carried out using powder X-ray diffraction (Bruker AXS-D8 diffractometer, Cu-K_α_ radiation (λ = 1.5406 Å); 2θ range 5–90°; scan speed of 1° min^−1^). The purity and stability of the as-prepared SnS_2_ nanoparticles were analyzed by FT-IR spectroscopy (Perkin Elmer Spectrum 2, Mumbai, Maharashtra, India) and thermal gravimetric analysis (EXSTAR TG/DTA instrument (Hyderabad, Telangana, India); heating rate 10°/min, ambient air atmosphere). Optical properties were investigated using a diffuse reflectance spectrophotometer (DRS) (Perkin Elmer, (Ahemdabad, Gujarat, India)) and photoluminescence (PL) spectrophotometer (Perkin Elmer Model, (Ahemdabad, Gujarat, India)) in the wavelength range of 200 nm to 800 nm. The morphology of SnS_2_ samples was analyzed using a field emission scanning electron microscope (Carl Zeiss, Bangalore, Karnataka, India) operating at 20 kV and equipped with an energy-dispersive X-ray analysis (EDXA, Bangalore, Karnataka, India) facility. For the FE-SEM analysis, the SnS_2_ powders were sprinkled on clean aluminum stubs using conducting carbon tape and were gold coated for 30 s using a sputtering unit.

## 3. Results and Discussion

### 3.1. Structure and Phase Analysis

**Figure 1:**
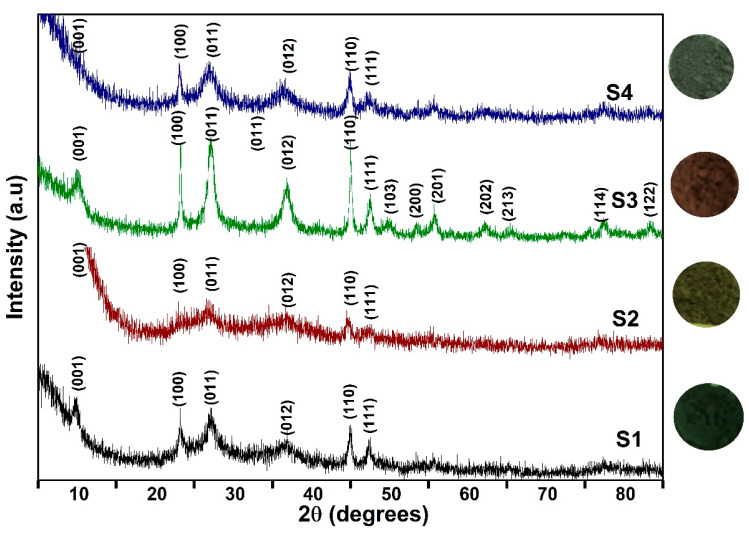
XRD plot of SnS_2_ nanoparticles synthesized in the absence and presence of capping agent by thermal decomposition method.

The XRD results for the SnS_2_ nanoparticles prepared via the thermal decomposition approach using different capping agents confirmed the formation of hexagonal SnS_2_ (JCPDS File No. 83-1705; Berndtite-2T phase) in all the samples (Figure 1). The XRD peaks at the 2θ values of 15.05°, 28.30°, 30.38°, 32.20°, 42.00°, 50.11°, and 52.63° were indexed to (001), (100), (002), (011), (012), (110), and (111) reflections of SnS_2_, respectively. It was observed that the XRD pattern for samples S1 and S3 exhibited sharp and well-defined peaks indicating high crystallinity and morphology characteristics that are discussed later in Section 3.4. The presence of two sets of peaks (sharp and broad) in the XRD patterns of S1 and S3 implied that the growth of certain facets was restrained, and that a special morphology was formed [18].

On the other hand, the XRD patterns of samples S2 and S4 exhibited broad and poorly defined peaks, indicating a low crystallinity of the nanoparticles. The crystallite size of SnS_2_ was calculated using the Debye–Scherrer formula. The crystallite size varied from 6.1 nm to 19 nm, as calculated using the (100) reflection, and 2.6 nm to 6.6 nm as calculated using the (011) reflection. The presence of the (001) peak in samples S1 and S3 indicated the formation of layered structures. The formation of highly crystalline nanoflakes assembled to form a flower-like structure for samples S1 and S2 was evident from the presence of sharp peaks in the XRD plot. In addition, the low intensity and poorly defined peaks in the XRD pattern pointed towards the formation of nanoparticles and nanoplates for samples S2 and S4.

### 3.2. Purity and Phase Stability

**Figure 2:**
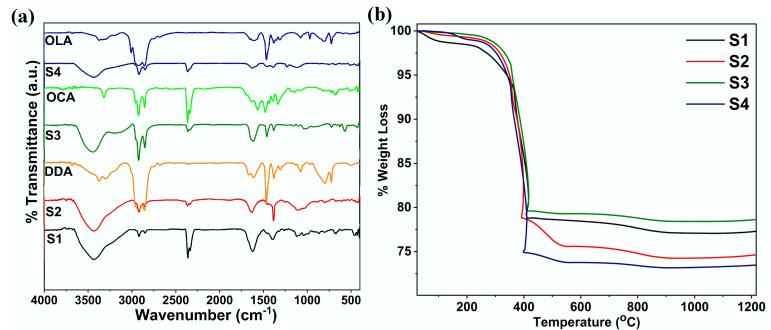
(**a**) IR and (**b**) TGA graph of SnS_2_ nanoparticles synthesized in the absence and presence of capping agent by thermal decomposition method.

The purity and phase stability of the as-prepared SnS_2_ samples were checked using FT-IR spectroscopy and thermogravimetric analysis (TGA), respectively. Figure 2a shows the FT-IR spectra of the SnS_2_ nanoparticles synthesized using different capping agents by the thermal decomposition method. The IR spectra of the SnS_2_ nanoparticles showed the presence of bands at around 3400 cm^−1^ and 1620 cm^−1^ attributed to stretching and bending vibrations of the hydroxyl group, indicating the presence of physisorbed moisture on the surface of the SnS_2_ nanoparticles [18]. The band at about 3200 cm^−1^ was attributed to N–H stretching, and the IR bands at around 2920 cm^−1^, 2840 cm^−1^, and 1390 cm^−1^ were ascribed to asymmetric and symmetric stretching and bending of the C–H group, respectively [30]. The bands at around 1260 cm^−1^, 870 cm^−1^, and 660 cm^−1^, were due to asymmetric and symmetric stretching and bending of the C–S group, respectively [31].

The IR spectra of all the SnS_2_ nanoparticles exhibited a band at around 680 cm^−1^ due to Sn–S stretching. The IR band at around 1110 cm^−1^ was ascribed to C–N stretching [18]. The assignments of IR peaks for all the SnS_2_ samples and capping agents used are listed in Table 2. The shift in the IR band positions confirmed the capping of nanoparticles with the different capping agents used (DDA, OCA, and OLA) on the surface of the SnS_2_ nanoparticles.

The TGA curves (Figure 2b) for all the SnS_2_ samples showed a small weight loss of ~1% at around 100 °C due to the loss of physisorbed moisture. The single-step weight loss at around 390 °C was attributed to the phase transformation (oxidation) of SnS_2_ to SnO_2_ (theoretical weight loss = 17.6%) [32]. The observed weight loss values for S1, S2, S3, and S4 were 19.8%, 20.6%, 20.4% and 24.1%, respectively. The SnS_2_ samples (S2, S3, and S4) prepared using capping agents exhibited marginally higher weight loss compared to those prepared in the absence of any capping agent (S1). This was attributed to the presence of more adsorbed organics on the surface of the SnS_2_ nanoparticles (S2, S3, and S4), indicating the presence of capping agents on the SnS_2_ nanoparticles. The TGA results were in agreement with the literature reports [18,32].

### 3.3. Optical Studies

**Figure 3:**
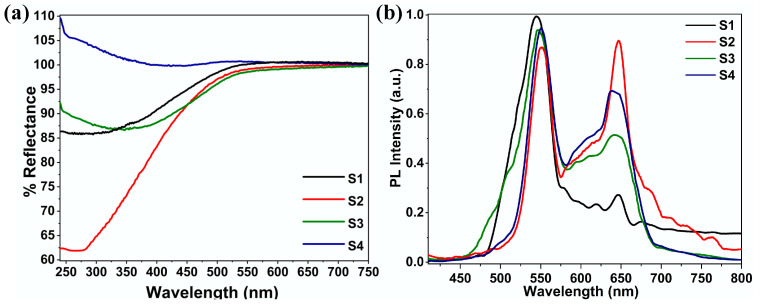
(**a**) DRS and (**b**) photoluminescence spectra of SnS_2_ nanoparticles synthesized in the absence and presence of capping agent by thermal decomposition method.

**Figure 4:**
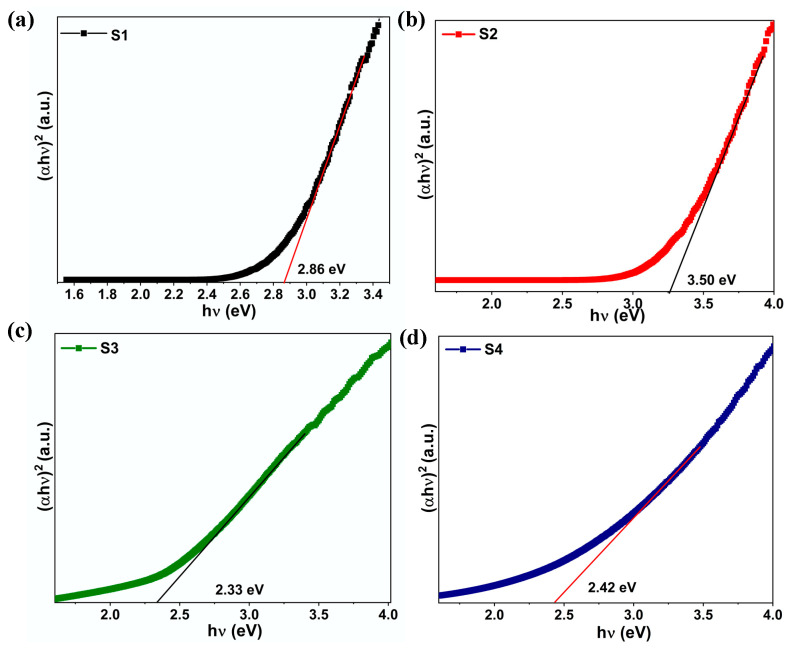
Tauc plot of SnS_2_ nanoparticles synthesized in the absence and presence of capping agent by thermal decomposition method.

The effect of the use of alkylamines on the optical properties of the SnS_2_ nanoparticles was investigated using DRS and PL spectroscopy. Figure 3 shows the DRS and PL spectra for the SnS_2_ nanoparticles. The DRS spectra exhibited band gap absorption for the SnS_2_ nanoparticles in the range of 400 nm to 550 nm. The PL spectra exhibited an excitonic emission at around 550 nm and a defect emission at around 645 nm. The excitonic emission exhibited a blue shift for samples S1, S3, and S4 with respect to sample S2. The PL results were in agreement with the DRS results. The band gap was estimated from the Tauc plots shown in Figure 4. The band gap was found to vary from 2.31 eV to 3.50 eV. The variation in band gap was attributed to the difference in crystallite size, with sample S1 with the smallest crystallite size exhibiting a band gap of 3.50 eV.

Further investigation of the PL spectra indicated an increased concentration of surface defects for samples S2, S3, and S4 when the capping agent was used during the synthesis. The presence of excess surface defects imparted novel characteristics and modifications to the existing properties [33]. The I_exc_/I_defects_ ratio was found to vary from 2 to 7.3 (Table 3). Sample S2 was observed to exhibit the highest amount of surface defects (I_exc_/I_defects_ = 7.3) and the smallest crystallite size. Alkylamines are reported to play multiple roles as solvent surfactants and reducing agents during the synthesis of nanocrystals [29]. The growth of crystals in the presence of alkylamines results in twinning and stacking during crystal growth resulting in internal defects and influencing the final properties of nanocrystals [34,35].

### 3.4. Morphological Analysis (FE-SEM and EDX Results)

**Figure 5:**
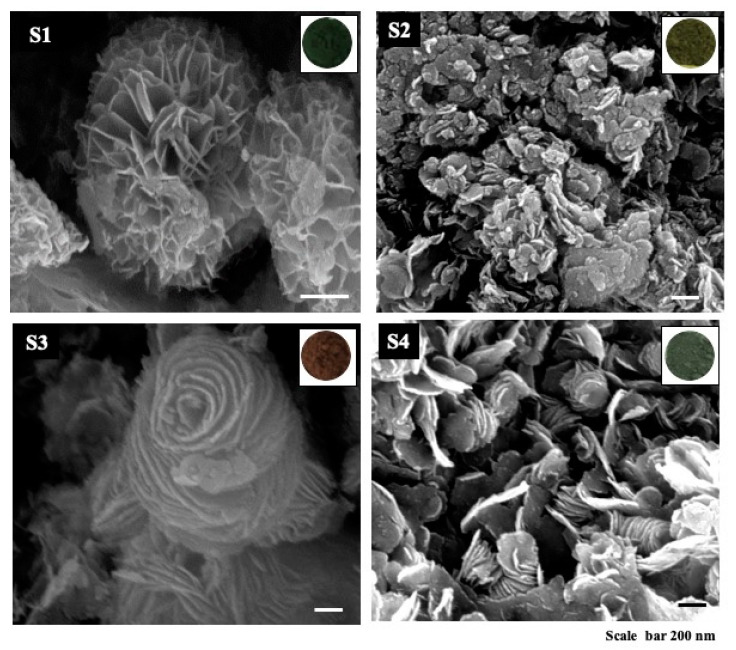
FE-SEM images of SnS_2_ nanoparticles (S1–S4) prepared using the thermal decomposition approach in the absence and presence of capping agents. The inset shows the digital images of powder samples.

The SnS_2_ nanoparticles synthesized using different capping agents by the thermal decomposition approach were characterized using FE-SEM analysis. Figure 5 shows the FE-SEM images of the SnS_2_ nanoparticles prepared in the absence and presence of capping agents such as octylamine, oleylamine, and dodecylamine. The presence of a capping agent played a vital role in influencing the morphology of the resulting nanoparticles. The FE-SEM analysis revealed the formation of a flower-like morphology for the pristine SnS_2_ nanoparticles (S1) prepared by the thermal decomposition approach in the absence of a capping agent. A web-like microstructure of SnS_2_ with a diameter of 800 nm was observed for Sample S1. The microstructures were formed by the assembly of flake-like nano-building units. The thickness of the flakes ranged from 10 nm to 15 nm.

On the other hand, the SnS_2_ nanoparticles synthesized in the presence of capping agents (octylamine, oleylamine, and dodecylamine) exhibited particle-like, twisted-flower-like, and stacked-plate-like morphology, respectively. Sample S2, prepared in the presence of octylamine showed the formation of irregular particles. Sample S3, prepared in the presence of oleylamine, showed the formation of rosette-flower-like morphology. A closer look at the FE-SEM images revealed that the flowers were formed by the twisting and wrapping of linear structures, assembled to form rosette-like structures with a rosette-like morphology with a diameter of 1.2 microns, and the thickness of the linear structure was observed to be around 40 nm. Sample S4, prepared in the presence of dodecylamine, showed the formation of a stack of plate-like structures, or nanoplates assembled into stacks. The diameter of a typical nanoplate was around 250 nm and the thickness were around 30–35 nm.

The composition of all the SnS_2_ samples (Sn:S) was analyzed using energy-dispersive X-ray analysis. The weight and atomic percent of Sn and S present in the SnS_2_ samples synthesized by the thermal decomposition method are given in Table 1. The EDXA results indicated the presence of tin and sulfur in all the samples, and the Sn:S ratio varied from 1:1.8 to 1:2.2, which was close to the theoretical value (1:2).

## 4. Mechanism for Morphology Evolution (Mechanism of Formation of SnS_2_ Nanoparticles with Different Morphologies)

**Scheme 1:**
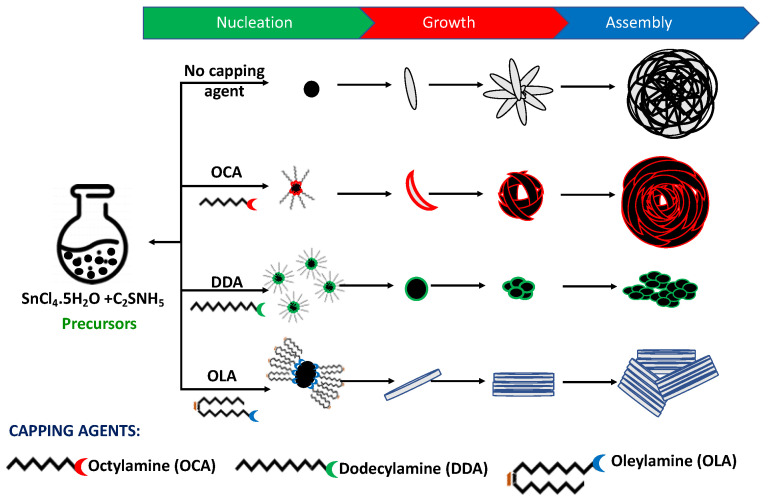
Proposed mechanism for the evolution of different morphologies of SnS_2_ prepared using thermal decomposition approach in the presence and absence of alkylamines.

Scheme 1 depicts the proposed mechanism for the formation of SnS_2_ nanoparticles with different morphologies using the thermal decomposition approach. The precursors (SnCl_4_·5H_2_O and CH_3_CSNH_2_ thioacetamide), when subjected to thermal decomposition, led to the formation of spherical SnS_2_ seeds in the initial stage of the reaction. As the reaction proceeded, the nuclei grew to form flakes and strands. The flakes/strands were the primary building blocks for the hierarchical structures and the flakes/strands assembled to form flower-like structures [18]. The presence of capping agents during the thermal decomposition played a vital role in controlling the morphology of SnS_2_ resulting in the formation of unique morphologies. Scheme 1 shows that the nuclei or basic building unit of SnS_2_ are arranged differently in presence of the different capping agents. The physicochemical properties of the capping different agents used during the synthesis decided the growth and assembly of the building blocks into nanostructures with a special morphology. The interaction of the surfactant molecule with the crystal seed drastically reduced the generation of nuclei. The reactant molecules then contributed to the characteristic growth of the nanocrystal.

The chain length of alkylamines also plays an important role in controlling the morphology of nanoparticles [21]. It is reported that higher activation energy and low reaction rate of amine with a longer carbon chain leads to the formation of smaller-sized quantum dots due to its higher capping capacity [36,37]. Hence, in this study, the growth was restricted, and this resulted in the formation of irregular particles or anisotropic crystals. Dodecylamine (C12) and oleylamine (C18), due to their longer alkyl(enyl) chains, resulted in the formation of irregular particles and stacks of nanoplates, respectively. On the other hand, octylamine (C8) resulted in the assembly of nuclei strands as a rosette-like morphology. Oleylamine (C18) acted as a surfactant and was adsorbed and subsequently passivated the surface, restricting further growth in planes, and resulting in the formation of nanoplates. The nanoplates were stacked together due to the interaction between the surfactant molecules adsorbed on the surface of the SnS_2_ nanoplates. Octylamine (C8) on the other hand, due to its high polarity, led to the fusion of nanoplates in a helical manner, resulting in a flower-like morphology. Long aliphatic chains of dodecylamine (C12) prevented the assembly and growth of nanoparticles and resulted in the formation of irregular particles.

## 5. Conclusions

SnS_2_ nanoparticles with different morphologies were successfully synthesized by the thermal decomposition of SnCl_4_·5H_2_O and thioacetamide in the presence of different surfactants (dodecylamine, octylamine, and oleylamine). The use of alkylamine during the synthesis affected the nucleation and crystal growth and had a great influence on the morphology and optical properties of the SnS_2_ nanoparticles. The SnS_2_ nanoparticles showed the assembly of flakes into flower-like nanostructures, nanoparticles, and nanoplates stacked together. The formation of characteristic structures had an influence not only on the structural features but also on the optical properties of the SnS_2_ nanoparticles. This approach was found to be beneficial for the surfactant-assisted synthesis of SnS_2_ nanoparticles with unique morphologies. Alkylamines, due to their multifunctional characteristics, were an integral part of the nanocrystal synthesis. The use of alkylamines during the synthesis not only tailored the morphology but also influenced the properties of the nanomaterials. Thus, it is imperative to explore mechanistic insights into the role of alkylamines in nanocrystal synthesis. The careful optimization of the reaction parameters such as solvents, temperature, surfactants, etc., resulted in nanocrystals with a controlled shape and size for applications in solar cells, catalysis, environmental remediation, etc.

## Figures and Tables

**Table 1:** Synthetic details, nomenclature, and morphology of all SnS_2_ samples.

Sample ID	Capping Agent	Color of the Product	Morphology	Crystallite Size		Sn:S Ratio (EDX)	Overall % Wt. Loss
				(100)	(011)		
S1	–	Dark Green	Flower-like	18.0	5.3	1:1.8	22.90
S2	Dodecylamine	Brown-green	Small nanoparticles	–	–	1:2.1	25.74
S3	Octylamine	Brown-green	Rosette-like morphology	31.5	8.8	1:2.2	21.6
S4	Oleylamine	Olive green	Nanoplates assembled into stacks	17.6	4.2	1:2.1	26.85

To investigate the role of alkylamines in detail, the synthesis was carried out by varying the amount of capping agent used. The results are discussed in Section 3.

**Table 2:** Assignments of IR peaks for the SnS_2_ samples and the alkylamines used as capping agents.

S1	S2	(DDA)	S3	(OCA)	S4	(OLA)	Band Assignments
3425	3424	3366	3443	3314	3443	3373 and 3300	υ_as_(NH_2_) and υ_s_(NH_2_)
3211	–	3292	–	2954	–	3005	δ (=C–H)
2920	2914	2923	2923	2923	2920	2950	υ_as_C–H
2847	2847	2855	2849	2852	2844	2852	υ_s_C–H
1619	1632	1611	1619	1620	1632	1611	δ (NH_2_)
–	–	1630	–	1638	1592	1620	δ (C–C)
–	–	1461	1461	1477	–	1461	
1392	1384	1378	1380	1388	1384	1376	δ (CH_2_)
1108	–	1303	–	1331	–	1307	
1097	1097	1073	1019	–	–	1070	δ (C–N)
–	–	966	–	–	–	966	δ (C–H) out of plane mode
–	–	801	–	–	–	801	
–	–	720	720	720	–	720	δ (C–C)
671	671	–	671	–	671	–	Sn–S

**Table 3:** PL peak positions, FWHM, intensity ratio (I_excitonic_/I_defects_), band gap, and crystallite size of SnS_2_ nanoparticles.

Sample ID	Peak Position (nm)		FWHM (nm)	(I_excitonic_/I_defects_)	Band Gap (eV)	Crystallite Size (nm)		Sn:S Ratio
	Excitonic Emission	Defect Emission				(100)	(011)	
S1	544	645	37.5	2	2.86	18.0	5.3	1:1.8
S2	551	645	36.9	7.3	3.50	6.9	2.6	1:2
S3	548	647	53.4	3.6	2.33	7.3	3.0	1:2
S4	551	645	40.7	2.4	2.42	10.3	2.9	1:2

## Data Availability

The data presented in this study are available on request from the corresponding author.
